# The effectiveness of ICT-based neurocognitive and psychosocial rehabilitation programmes in people with mild dementia and mild cognitive impairment using GRADIOR and ehcoBUTLER: study protocol for a randomised controlled trial

**DOI:** 10.1186/s13063-017-2371-z

**Published:** 2018-02-12

**Authors:** Martina Vanova, Eider Irazoki, J. Antonio García-Casal, Fernando Martínez-Abad, Cristina Botella, Kate R. Shiells, Manuel A. Franco-Martín

**Affiliations:** 1 Institute of Biomedical Research, University of Salamanca, Salamanca, Spain; 2 Faculty of Psychology, University of Salamanca, Salamanca, Spain; 3 Faculty of Education, University of Salamanca, Salamanca, Spain; 4 Department of Basic and Clinical Psychology and Psychobiology, Universitat Jaume I, Castellón, Spain; 5 Centre of Gerontology, Faculty of Humanities, Charles University, Prague, Czech Republic; 6 INTRAS Foundation, Ctra de la Hiniesta 137, 49024 Zamora, Spain; 7 Ciber Fisiopatología Obesidad y Nutrición, CB06/03 Instituto Salud Carlos III, Madrid, Spain

**Keywords:** Dementia, Alzheimer’s disease, MCI, Cognitive rehabilitation, Cognitive intervention, Computer-based intervention, Psychosocial intervention, Cognition

## Abstract

**Background:**

Cognitive rehabilitation is a highly individualised, non-pharmacological intervention for people with mild cognitive impairment (MCI) and dementia, which in recent years has also been developed for various IT platforms.

**Methods:**

In this study, we aim to evaluate the effectiveness of the cognitive rehabilitation software GRADIOR in a multi-centre, single-blinded randomised controlled trial with people with MCI and mild dementia. A total of 400 people with MCI and mild dementia will be randomly allocated to one of four groups. This trial will compare the cognitive rehabilitation treatment using the GRADIOR programme with a psychosocial stimulation intervention (PSS) using the ehcoBUTLER platform, with a combined treatment consisting of GRADIOR and ehcoBUTLER, and with a group receiving treatment as usual during a period of 1 year.

**Discussion:**

The outcomes of this clinical trial will be to determine any relevant changes in cognition, mood, quality of life, activities of daily living and quality of patient-carer relationship after 4 months and 1 year of intervention in a cross-sectional group comparison. Participants will be followed-up for 1 year to investigate potential long-term effects of the conducted treatments.

**Trial registration:**

Current Controlled Trials ISRCTN, ID: 15742788. Registered on 12 June 2017.

**Electronic supplementary material:**

The online version of this article (doi:10.1186/s13063-017-2371-z) contains supplementary material, which is available to authorized users.

## Background

Current prevalence rates of dementia in Spain are approximately 1.75% of the state population, which equates to more than 800,000 people [1]. In their annual report, Alzheimer’s Disease International [2] estimate a prevalence of 1.6 million new cases of dementia per year in people over 60 years of age solely in Western European countries, which is accompanied by increasing costs per capita each year. Mild cognitive impairment (MCI) is a condition with various subtypes of different progression and prognoses, with 20–40% of cases progressing to dementia [3]. The overall crude prevalence of MCI is estimated at around 6–12% at the lower end, with some reviews reporting a prevalence rate of up to 42% in people over 60 years of age [4]. Progressive cognitive deficit, which is present in dementia, restricts the individual independence and the activities of daily living of every person affected [5, 6]. Moreover, it also places high demands on caregivers, family members and health professionals involved in their care [7]. For these reasons, it is important to find a sustainable approach to maintain the independence and self-efficacy of people with dementia (PwD) and MCI. Importantly, cognitive interventions seem to be beneficial not only for PwD, but also for their caregivers [8].

Cognitive rehabilitation is a highly individualised intervention specific to patients’ needs and goals [9], and in people with mild dementia is used to improve or maintain their cognitive status [10]. On the other hand, cognitive trainings are focussed on training or restoration of one particular ability or skill (e.g. working memory, attention, etc.) [11]. Cognitive rehabilitation has shown to be more effective than cognitive training or group reminiscence therapy, having a positive effect on decreasing the functional decline of PwD [12]. Similarly, a systematic review by Clare and Woods reported no significant effects of cognitive training interventions; while the only study on cognitive rehabilitation included in the review found preliminary but promising results of this approach when applied in people with Alzheimer’s disease and vascular dementia [11]. Intensive cognitive rehabilitation programmes can also reduce behavioural disturbances in PwD [13], but their influence on activities of daily living, quality of life or symptoms of depression remains inconclusive [14]. Cognitive rehabilitation can also significantly improve cognition, and reduce behavioural disturbances and symptoms of depression in MCI [15]. Furthermore, cognitive rehabilitation has been proven to have a positive effect in caregivers of PwD by lowering the feeling of burden and improving their confidence in caregiving situations [16].

Computer-based cognitive interventions are a cost-effective and flexible approach which are nowadays easily accessible to the public [17]. Computer-based cognitive trainings have shown to be efficient in people with MCI, which is important when addressing Alzheimer’s disease prevention or delaying the onset of the disease [18]. Currently, the field of cognitive rehabilitation for people with MCI is relatively underdeveloped in comparison to cognitive training. However, studies have shown that computer-based cognitive rehabilitation can also improve general cognition [19], as well as reduce depression and anxiety symptoms in MCI [20]. A meta-analysis by García-Casal et al. [21] reported a significant improvement in cognition, depression and anxiety in PwD involved in computer-based cognitive interventions. Furthermore, it has been reported that computer-based cognitive rehabilitation is able to delay memory deterioration in PwD [22]. People with dementia with low levels of function can also benefit from computer-based rehabilitation programmes which can improve different cognitive domains and mood [23]. Although a different meta-analysis did not reveal any significant changes in cognition for PwD, more studies are necessary in order to discover the potential long-term effects of computer-based cognitive interventions in PwD [24].

Stimulating social activities can serve as an effective prevention strategy in primary care, delaying cognitive deterioration in older PwD and MCI [25]. Psychosocial interventions can also introduce positive changes in cognition, mood or communication in PwD [26] and MCI [27].

GRADIOR is a computer-based programme used for the neuropsychological rehabilitation of people with various cognitive impairments of different aetiology, as well as for cognitive stimulation of healthy individuals. GRADIOR consists of dynamic exercises designed to stimulate all cognitive abilities, offering tools to tailor the cognitive intervention to each person according to their individual needs. The programme also provides tools for the evaluation of the current performance in every cognitive domain, which is important for treatment adjustments (changing difficulty levels for each task independently according to the participants’ performance).

The psychosocial stimulation platform ehcoBUTLER was designed to improve the quality of life of older people and their caregivers by promoting a healthy lifestyle and stimulating active aging through the use of tools that enhance positive emotions and cognitive training [28]. EhcoBUTLER contains leisure activities and other activities focussed on care, and can be adjusted to the needs of every person individually. The aim of all activities is to reduce social isolation, cognitive and physical inactivity and sedentary behaviours, and to promote health, satisfaction and personal wellbeing.

The platform has different versions. The leisure version contains various applications including Wellbeing (Walk), Internet, Book of Life, My Memories (photographs, music and videos), Leisure Activities (books and puzzles) and Social Applications (friends, mail and videoconference). The care version adds activities to each module, such us Nutrition and Lifestyle recommendations, which is part of the Wellbeing module. The platform also facilitates communication and can be used by families and caregivers with the aim of sharing information and keeping up to date with the daily activities or conditions of people. The platform can be used in different devices such as PCs, touch-screen computers and tablets.

Currently, despite the large number of different computer-based cognitive rehabilitation programmes, there is a lack of large-scale randomised controlled trials (RCTs) on individualised cognitive rehabilitation programmes [12]. Furthermore, despite providing initially promising results, the results on the effectiveness of cognitive rehabilitation trainings are still inconclusive and more RCTs are required in this area [29].

For this reason, we will conduct an interviewer-blinded, multi-centre randomised controlled trial with four parallel groups. The primary aim of which is to determine the effectiveness of the cognitive rehabilitation programme GRADIOR and the psychosocial stimulation platform ehcoBUTLER, separately or in a combined treatment, on the cognition, quality of life, activities of daily living, relationships with carers and depressive symptoms of people with MCI and PwD. Based on previous research, we hypothesise that people with MCI and PwD in the cognitive rehabilitation group using GRADIOR or combined treatment will improve their cognition in comparison to other treatment groups and/or the treatment as usual (TAU) group. A secondary aim is to examine the interaction between psychosocial stimulation and cognitive rehabilitation in order to discover their synergies or usefulness. The duration of the RCT is 1 year of treatment followed by a 1-year follow-up study.

A preliminary study on the usability of GRADIOR and ehcoBUTLER will be conducted through focus groups and users’ testing in order to identify any issues in the programme’s features which may potentially influence its effectiveness in MCI and PwD. Additional data will be collected and any incidents will be monitored to improve the final usability of both programmes.

## Methods

### Design

This is a multi-centre, randomised controlled, interviewer-blinded trial, with four parallel groups, 1:1:1:1 allocation ratio. Potential participants will be identified by collaborating professionals at recruitment sites and those interested in participating will sign an informed consent form. Subsequently, those selected based on inclusion/exclusion criteria (*n* = 400) will undergo preliminary testing and will be randomly allocated to one of the four study groups. In this study, there are three intervention groups and one group receiving TAU. Intervention groups are: (1) cognitive rehabilitation with the GRADIOR programme (GRA), (2) a computer-based psychosocial stimulation group (ehcoBUTLER) (PSS) and (3) a group receiving a combined treatment of GRADIOR together with computer-based psychosocial stimulation (ehcoBUTLER CARE) (COM). Primary and secondary measures data will be collected at baseline, at 4 months after the start of the treatment and at 12 months, immediately after completing the interventions. Follow-up assessments will be carried out 4 and 12 months after finishing the treatment (months 16 and 24 of the study). A preliminary study of usability will be conducted before the start of the trial. The full study timeline is described in Table 2.

### Sample size

Maximal sample size for the study was set at 400 participants with dementia and MCI. We considered a 15% dropout by participants during the 12 months of interventions and 12 months of follow-up. Therefore, the final sample size for the study is likely to be 340 participants.

Considering that the standard deviation of the selected population in the selected variables of the study was unknown and the population size was undetermined, we opted for a calculation of the sample size for an infinite population with the maximal level of variability of the dependent variables of the study (*p* = *q* = .5). If the population scattering parameters are unknown, this calculation allows for a more conservative sample size, meaning the biggest sample size [30].

The calculation was executed using the following equation (α = .05):

$$ n=\frac{Z_{\raisebox{1ex}{$\propto $}\!\left/ \!\raisebox{-1ex}{$2$}\right.}^2\ast {0.5}^2}{e^2}, $$

where 0.52 is a maximal variability (*p* * (1 − *p*)), *Z*_∞/2_ corresponds to scores in the percentile *α*/2 (.025) of a normal distribution, and *e* corresponds to the error acquired for the desired sample size, with a result of *e* = 5.3% for a sample size of 340 participants.

On the other hand, based on the Friedman table for determining effect size [31], for the primary outcome measure ADASCog to achieve 80% power (alpha 0.05) on a sample of 340 participants we are looking for an approximate effect size of 0.30, which is similar to a separate study protocol on cognitive rehabilitation in PwD [9].

### Participants

The initial identification of suitable participants with MCI and mild dementia will take place at various sites within the province of Zamora, Spain: community centres, memory clinics, public hospitals, daycare centres and residential centres.

The inclusion criteria for participants with MCI and mild dementia will be: aged over 60 years, possess the capacity for making decisions, have a reference person or a caregiver, and be able to read and write in Spanish. For those with a diagnosis of MCI, the study will comply with the criteria of Petersen [32] and the International Working Group (IWG) for people with amnestic MCI [33], where participants should have a Clinical Dementia Rating (CDR) score = .5 and Geriatric Depression Scale (GDS) < 5. For those with a diagnosis of dementia, participants should meet *the Diagnostic and Statistical Manual for Mental Disorders* (DSM-5) diagnostic criteria for major neurocognitive disorders, CDR score ≥ 1 and < 2 and GDS score < 5 [34].

The exclusion criteria for both groups will be: loss of senses that make it difficult to use the devices (blindness, deafness) or severe physical comorbidity (a disease causing tremor), current significant neurological disease (e.g. Parkinson’s disease, Huntington’s disease, Lewy-body dementia, epilepsy, multiple sclerosis, traumatic brain injury, etc.), current significant anxiety or depressive disorder (DSM-5 criteria) that would affect cognitive functioning and thus act as confounders, having a current history of alcoholism and/or substance abuse (including alcohol-related dementia), or currently taking any antipsychotic medications. All participants must be willing to participate in the study voluntarily and will sign an informed consent form. Some of the participants may be taking anticholinesterase inhibitors or memantine. These participants must have been stabilised on their current dose for a minimum of 1 month prior to the baseline assessment. This is to ensure that any changes will not be confounded by medication effects. All inclusion/exclusion criteria are summarised in Table 1.

**Table 1:** Inclusion/exclusion criteria

	MCI	PwD
Inclusion criteria	Age 60 + years Reference person Reads and writes in Spanish Petersen criteria (1999) for PwaMCI: (1) memory problems, (2) objective memory disorder, (3) absence of other cognitive disorders or repercussions on daily life, (4) normal general cognitive function and (5) absence of dementia CDR = .5 GDS < 5 MMSE ≤ 27	Age 60 + years Reference person Reads and writes in Spanish DSM-5 criteria for mayor neurocognitive disorder: A. Evidence of significant cognitive decline from a previous level of performance in one or more cognitive domains: complex attention, executive function, learning and memory, language, perceptual-motor, social cognition. (1) Concern of the individual, a knowledgeable informant or clinician that there has been a significant decline in cognitive function, (2) A substantial impairment in cognitive performance documented by standardised neuropsychological testing B. The cognitive deficits interfere with independence in everyday activities. At a minimum, assistance should be required with complex instrumental activities of daily living, such as paying bills or managing medications C. The cognitive deficits do not occur exclusively in the context of a delirium D. The cognitive deficits are not better explained by another mental disorder, with or without behavioural disturbance (e.g. major depressive disorder, schizophrenia) CDR ≥ 1 and < 2 GDS < 5 MMSE 20 < x < 25
Exclusion criteria	Loss of senses Severe physical comorbidity Current significant neurological disorder Current significant anxiety or depressive disorder History of alcoholism and/or substance abuse Current antipsychotic medication and/or cognition-affecting medication Recent hospitalisation for mental illness or stroke (in the past 6 months)	Loss of senses Severe physical comorbidity Current significant neurological disorder Current significant anxiety or depressive disorder History of alcoholism and/or substance abuse Current antipsychotic medication and/or cognition affecting-medication Recent hospitalisation for mental illness or stroke (in the past 6 months)

*MCI* mild cognitive impairment, *PwD* people with dementia

An additional criterion will be MMSE score, defined by a cut-off point of ≤ 27 points for MCI and 20 < x < 25 points for people with mild dementia. The score of each participant in this test will be adjusted according to their age and years of education based on the study by Blesa, Pujol and Aguilar [35] as follows: (1) for participants between 51 and 75 years of age and 8 or less years of education + 1 point, and − 1 point for more than 17 years of education; (2) for participants over 75 years of age and 8 or less years of education + 2 points, and + 1 point for 9–17 years of education.

### Allocation and randomisation

Participants will be randomly allocated in a 1:1:1:1 ratio to either of the four trial arms using a computer-generated random number sequence. The Epidat 4.1 programme will be used to generate this sequence. The allocation will be carried out by an independent researcher who will be unaware of the characteristics of the study. Participants will be allocated to each group using simple randomisation. Simple randomisation will allocate every participant randomly in a group independently of their characteristics (age, sex, education, area, etc.).

Participants will agree to participate before the random allocation takes place and without knowing to which group they will be allocated. The staff member responsible for sequence generation will provide participants with the details of the treatment group that they were allocated to independently from study coordinators. Participants will receive a sealed envelope by post with sufficient details and instructions about how to start their participation in the treatment.

### Procedure and intervention

#### Usability study

The techniques used to carry out the usability study will be: focus group, users’ testing and usability questionnaires.

The usability study will consist of three different phases:

1. Pre-experimental phase

### Focus group

Focus group of GRADIOR: this will be used to determine whether the experience of using the program is adequate and if its interface is functional and intuitive for people with MCI and mild dementia, as well as for the healthcare professionals. The following participants will be recruited: two groups with MCI, two groups with mild dementia and two groups of professionals who have experience with GRADIOR in PwD and/or MCI.

Focus group of ehcoBUTLER: this will be used to determine whether the characteristics and tools of the ehcoBUTLER platform, as well as its design, would be well accepted by people with MCI and mild dementia, as well as by relatives and professionals. Participants will be shown a video about the programme and its tools. The following participants will be recruited: two groups with MCI, two groups with mild dementia, two groups of informal carers or family members and two groups of professionals who have worked or work with PwD and/or MCI.

The different focus groups of both GRADIOR and ehcoBUTLER will consist of five to six people each. In order to carry out focus groups, scripts with general and specific open questions will be developed to obtain the necessary information for the research. In addition, participants will obtain an informed consent to record the sessions in audio and video for later analysis.

### Users’ testing

This will be carried out in a usability laboratory and users’ interactions with the GRADIOR and ehcoBUTLER programs will be recorded.

GRADIOR user test: five people with MCI, five with mild dementia and five professionals will be recruited who have no prior experience with GRADIOR.

ehcoBUTLER user test: five people with MCI, five people mild dementia and five professionals will be recruited.

In both cases, the users will perform a series of practical tasks in the GRADIOR and ehcoBUTLER programs.

Only one user at a time can perform the experiment in order to establish a careful monitoring of their actions and behaviour; measures of effectiveness, efficiency and satisfaction. In addition, the session will be recorded in audio and video for later analysis. All participants should have some prior experience with information and communication technology (ICT). Before starting the tests, participants will be asked to sign an informed consent. At the end of the assignment the participants will have to complete the System Usability Scale (SUS), which will serve to assess the usability and satisfaction with the programs.

2. Experimental phase

During this phase, data in the RCT will be collected from the participants using GRADIOR and/or ehcoBUTLER about all the incidents that occurred with the GRADIOR and ehcoBUTLER programs for further analysis. All the incidents that arise during the treatment about technical aspects and use of the program, referring to both the software and hardware, will also be collected. These will be reported on a record sheet. This data will be analysed and will serve to assess the problems encountered by users and the difficulties of implementing the program.

3. Post-experimental phase

Once the experiment is finished, the User Experience Questionnaire (UEQ) will be administered, which will serve to assess the participants’ user experience.

### Intervention

Intervention sites will vary depending on accessibility and mobility of participants. Community centres, social care centres, memory clinics, community centres, residential care homes and similar facilities within the province will participate in the study.

The first cognitive rehabilitation session with GRADIOR will be used as a baseline which will help determine each participant’s level of ability. The baseline treatment is a shorter version of the usual intervention and includes exercises from various subdomains which will determine the level of difficulty for the main treatment and also help the participants to familiarise themselves with the programme and its interface. During the first psychosocial stimulation session with ehcoBUTLER, a member of staff will create a user profile for each participant and demonstrate how to use the platform.

#### GRADIOR: Computer-based cognitive rehabilitation (GRA)

GRADIOR is a neuropsychological rehabilitation programme activating seven areas of cognitive functions (memory, attention, orientation, perception, calculus, executive functioning and reasoning) with specific tasks. In total, GRADIOR contains 48 different types of exercises with 2 to 11 levels of difficulty (see Appendix). For every participant, the GRADIOR treatment will include exercises for all cognitive subdomains. At the starting point of the treatment, all the participants will receive the same set of exercises which will differ in the level of difficulty for every subdomain based on the participant’s performance in the preliminary baseline treatment.

Participants will attend treatment sessions three to four times per week for 30 min per session. The days or distribution of treatment sessions during the week will depend on the availability of each centre and on the individual availability of participants. The treatment for each participant will be adjusted after 4 months when a midterm assessment will take place. The treatment can also potentially be adjusted before this time based on the participant’s performance in the exercises.

Every exercise in GRADIOR has a duration of 1 min during which different stimuli are presented to the participant who is then asked to provide a specific response (e.g. point only to a certain group of objects). During one treatment session, the exercises are randomised for each subdomain; however, the order of the subdomains in the treatment is fixed. If the participant does not complete all exercises on the list in one session, the subsequent session will pick up from where they stopped, i.e. with tasks for subdomains which are next on the list. In this way, the programme continues in a loop. The user does not repeat the same exercises every session, but only once they have finished the previous set.

#### ehcoBUTLER: Computer-based psychosocial stimulation (PSS)

EhcoBUTLER is an e-health platform available via the Internet, which aims to promote physical and mental health, social participation, quality of life and personal wellbeing. EhcoBUTLER is aimed at people with MCI or mild dementia as well as their carers.

During the first two sessions participants will be trained to use the system, after which they will use the platform for 5 h per week or more. The time schedule of usage of the platform will depend on each participant and their needs and individual availability. It will be recommended to participants that they use the platform for 2 h per day. To ensure correct usage of the platform, the sessions taking place outside of the participants’ homes will be facilitated by a member of staff. Study coordinators will have access to the data registered by the platform to ensure the participation of all users. On the other hand, the therapist may use the ‘Task List’ function to propose activities to the participants and subsequently monitor their accomplishments.

#### ehcoBUTLER CARE: Combined treatment (GRADIOR + psychosocial stimulation) (COM)

The group receiving both cognitive rehabilitation and the psychosocial stimulation treatment will have scheduled sessions with GRADIOR twice per week for 30 min per session and will be advised to use ehcoBUTLER for at least 3 h per week. The treatment sessions with GRADIOR will be the same as the group only using GRADIOR and the same procedure will be followed to determine the baseline, adjustments of treatments and levels of difficulty.

#### Treatment as usual

Treatment as usual (TAU) is defined as individual care depending on each participant’s needs, including medication, social activities, etc. which will not be influenced by study coordinators or other staff members included in the study. TAU is usually region-specific due to influences in a person’s access to services and treatment availability. Therefore, the outcomes of care for these participants may vary. Any cognitive intervention, psychosocial stimulation programmes or activities will be recorded as potential influences on the outcome measures. Participants in this group will be put on a waiting list for the cognitive rehabilitation treatment with GRADIOR and ehcoBUTLER, and will be offered these treatment choices once the main trial is finished. TAU is usually also available to participants in the active treatment groups.

### Interventions – modifications

The intermediate testing of cognitive functions, screening, quality of life and evaluation of emotional state scheduled after 4 months will provide additional data for treatment modifications. These tests are important in case of a scenario where there is a lack of efficacy of the treatment, observed harms or changes in physical health with an impact on cognition and emotions, or for any other relevant reason.

### Treatment adherence

Interviewers will be trained clinical psychologists or neuropsychologists. All interviewers and staff included in the study will be provided with a set of guidelines for communication with participants to ensure that they have a correct understanding of the treatment sessions and that effective communication is established between participants and all staff members. During all GRADIOR treatment sessions, a member of staff will be present to provide support to participants, enabling their full participation in the treatment. However, the member of staff will not interfere with the treatment. All participants will be given contact details and times when they can contact study coordinators regarding any difficulties experienced or if questions arise during the treatment.

The sessions for both groups receiving cognitive rehabilitation treatment (GRADIOR and ehcoBUTLER CARE or combined treatment) will be managed by two study coordinators independently in each group to avoid personal bias. The treatment coordinators are trained in advanced in how to use the GRADIOR programme, including setting up and adjusting treatments, and also have a degree in psychology. Participants receiving treatment in a group setting will be supported during each session by a separate staff member independent from the treatment coordinators.

To ensure a high number of participants finish the study and participate in as many treatment and evaluation sessions as possible, they will be offered different dates of evaluation. The data collected from participants who prematurely finish the study will be used for partial data analysis. Interviewers involved in assessment and data collection will be trained centrally in trial requirements, ensuring that they have full knowledge of all measurement scales and know how to correctly administer tests. All interviewers will also receive training in the outcome measures used during the trial and will comply with the same administration criteria and standards for every scale and measurement used. Interviewers will have access to video guidelines for the correct administration and scoring of all tests used during the study. During training, interviewers will learn how to administer each test used in the study following the video guidelines. Subsequently, to ensure correct scoring, interviewers will have to score a tested person featured in the video with a requirement of inter-rater reliability of *κ* = 0.80 (between the interviewer in the training and the interviewer from the video). Study coordinators will be responsible for correct data collection and administration of all tests by interviewers.

### Resources

The RCT relies on human resources and materials available in the INTRAS Foundation memory clinics in Zamora and Valladolid, Spain, and is supported by the Department of Innovation at the Iberian Institute of Research in Psychosciences (IBIP) in Zamora, Spain and the Department of Development at the INTRAS Foundation in Valladolid, Spain. The IBIP provides access to neuropsychological tests, infrastructure, materials and computers, which are necessary for conducting the study.

The centres involved in the study which do not belong to INTRAS Foundation will independently provide materials and human resources necessary for the study. The INTRAS Foundation will provide the centres with the GRADIOR software, access to ehcoBUTLER platform, training of the interviewers and supervision during the interventions to register any incidents or potential problems.

The other collaborating centres are the University of Salamanca, the Study Institute of Health Sciences of Castile and Leon health system (IECSCYL) and the Psychiatry Services and Mental Health Department of the Care Services in Zamora, Spain.

### Ethical approval

The protocol was submitted for ethical approval in May 2017 to the Zamora Provincial Hospital Ethics Committee and the trial was registered with ISRCTN with a reference number 15742788. Ethical approval was obtained on 17 May 2017. All participants will obtain written informed consent about their rights to access the information, data modification, elimination or correction following the Ley 11/1999 for data protection. All participants will sign the informed consent form at the beginning of the study and before any procedure is conducted.

Up until now, no adverse effects of GRADIOR or ehcoBUTLER have been reported and there are no known side-effects related to the usage of these programmes. Should any adverse effects be reported during the conduct of the study, they will be recorded by the staff member involved and the appropriate study coordinator will be informed.

### Outcome measures and hypotheses

#### Primary outcome measures

GRADIOR stimulates cognitive functioning, which is reflected in changes in separate cognitive domains. Outcome measures were selected accordingly. The primary aim of the study is to determine whether the cognitive rehabilitation programme GRADIOR improves cognition of people with MCI and PwD in a specific (GRA) or combined treatment group (COM). Based on previous research in this area, and our previous experience with the GRADIOR programme in a clinical setting, we expect that the two treatment groups using GRADIOR solely and in combination (COM) will achieve significantly higher scores in cognitive testing than the TAU group after 4 and 12 months of treatment. The primary outcome measure will be an ADASCog Cognitive Subscale [36].

The change in cognitive performance after a period of 4 months and 12 months in comparison with baseline results will be measured in all study groups using the *ADASCog Cognitive Subscale* [36] as the primary outcome, which evaluates cognitive performance in memory, praxis, orientation and language. Additionally, the Spanish version of the *Mini-Mental State Examination (MMSE)*, consisting of 35 questions [37], will be used*.* For a more comprehensive evaluation of a participant’s cognitive profile, we will use a variety of tests: the *Clock Drawing Test* [38] for general cognitive and spatial functioning, *WAIS III – Coding digit symbol, Calculus and Digit Span* subtests [39] to evaluate processing speed, numeric reasoning and working memory respectively, the *Rivermead pattern recognition test* [40] for visual memory, *Trail-making tests A and B* [41] to monitor motor functioning, executive functioning and visual-spatial abilities (part A) and also sustained attention (part B), the *Cambridge Cognitive Examination (CAMCog) – matrices* subtest [42] to examine intellectual ability, analogic reasoning and abstraction capacity and *Semantic and phonologic verbal fluency* [43] and [44] on animal category and letters P, M and R.

#### Secondary outcome measures

Changes in quality of life (QoL) scores will be measured using the *EuroQol EQ5D-5 L* [45] which assesses quality of life from five dimensions, and the *Luben Social Network Scale (LSNS)* [46] which looks at social engagement, including relationships with family and friends. QoL scores will be compared between all the intervention groups and with the control group to determine which intervention obtains the best results and most influences the outcome. We will compare scores for each intervention group at 4 and 12 months in order to determine at which point in the treatment the various interventions most influenced quality of life. All participants will be assessed and compared using the *Geriatric Depression Scale (GDS)* [47], a 15-item version of a self-rating scale, in order to determine which type of intervention, if any, has an influence on depressive symptoms in PwD. Demographic data, activities of daily living (instrumental – IADL and non-instrumental – ADL), functional aspects and cost-effectiveness will be assessed using the *InterRAI Home Care* [48]. The quality of the relationship between the participants and their carers will be assessed using the *Quality of Carer-patient Relationship (QCPR) questionnaire* [49].

As GRADIOR focuses solely on cognitive rehabilitation, we assume that those participants with dementia receiving only the GRADIOR treatment will not see improvements in their quality of life, depressive symptoms, the quality of their relationships with a carer and/or activities of daily living after 4 or 12 months.

At the beginning of the study, participants will also receive the *Everyday Technology Use Questionnaire (ETUQ)* [50]. This will assess their perceptions about technology, difficulties encountered when using technology, and their degree of experience with technology that might be relevant for the study.

Carers will also be assessed at the same time as participants, whereby we will evaluate the following: their anxiety using the *State-Trait Anxiety Inventory (STAI)* [51], symptoms of depression with the *Beck Depression Inventory II (BDI-II)* [52], carer overload using the *Caregiver Burden Interview – Zarit Test* [53] and the quality of their relationships using the *Quality of Carer-patient Relationship (QCPR*) *questionnaire* [49]. We anticipate that GRADIOR will positively influence carer-outcome measures, and therefore, we hypothesise that in both intervention groups using GRADIOR, caregiver burden, anxiety and depression will decrease, and the relationship between the carer and the PwD will improve.

The data collected from the InterRAI Home Care Assessment will be used to analyse the cost-effectiveness of the treatment and its impact on health. The economic evaluation will employ a cost-consequences framework evaluating the associated costs and outcomes of the three types of interventions, in comparison to usual care. The evaluation will also assess the main sources of costs associated with the interventions. Primary analysis will be based on the most plausible real-world costs, including the cost of the technology and technical progress. The support costs for GRADIOR and ehcoBUTLER associated with technical aspects of installation, maintenance of hardware and information, as well as communication technology input related to software and data management, will be based on data gathered from sites. This will also include the input of professionals for guiding participants and support provided to participants throughout the intervention. For a complete list of the assessment tests used and the time schedule see Tables 2 and 3 respectively.

**Table 2:** Assessments

Activity/assessment	Staff member	Time	Pre-study	Pre-study baseline	2	4	6	8	10	12	16	24
Usability study	Interviewer	N/A	X									
Focus group		90										
SUS		N/A										
Consent Form	Study coordinator	5	X	X		X				X	X	X
Inclusion/exclusion criteria	Study coordinator	N/A	X									
DSM-5 criteria	Interviewer	N/A	X									
Cognitive assessment	Interviewer	106										
MMSE		20		X		X				X	X	X
ADASCog		40		X		X				X	X	X
WAIS III: Calculus		5		X		X				X	X	X
WAIS III: Digit Span		5		X		X				X	X	X
WAIS III: Coding digit symbol		2		X		X				X	X	X
TMT A and B		10		X		X				X	X	X
Semantic and phonologic verbal fluency		4		X		X				X	X	X
CAMCog matrices		5		X		X				X	X	X
Clock Drawing Test		10		X		X				X	X	X
Pattern recognition test		5		X		X				X	X	X
ADL/iADL (InterRAI HC)	Interviewer	N/A		X		X				X	X	X
Quality of life	Interviewer	20										
EQ5D-5 L		5		X		X				X	X	X
LSNS (Luben)		15		X		X				X	X	X
Mood	Interviewer	15										
GDS		15		X		X				X	X	X
Patient-carer relationship		N/A										
QCPR				X		X				X	X	X
Technological assessment	Interviewer	N/A										
ETUQ				X								
Randomisation	Study coordinator	N/A		X								
Treatment (group-specific)	Study coordinator	N/A			X	X	X	X	X	X		
Termination Form	Study coordinator	N/A	As needed throughout the study									
Adverse Event Form	Study coordinator	N/A	As needed throughout the study									
Progress notes	All	N/A	X	X		X				X		
Communication log	All	N/A	Every phone or in-person contact outside of regular visit									

*ADASCog* Alzheimer’s Disease Assessment Scale – Cognitive Subscale, *ADL* activities of daily living, *CAMCog* Cambridge Cognition Examination, *EQ5d-5 L* EuroQoL 5 dimensions, 5 levels, *ETUQ* Everyday Technology Use Questionnaire, *GDS* Geriatric Depression Scale, *LSNS* Luben Social Network Scale, *MMSE* Mini Mental State Examination, *N*/*A* not applicable, *QCPR* Quality of Patient-carer Relationship, *SUS* System Usability Scale, *TMT* Trail-making test

**Table 3:** Time schedule

Activity/month	Responsible	−3	−2	−1	0	1	2	3	4	5	6	7	8	9	10	11	12	13	14	15	16	17	18	19	20	21	22	23	24	25	26	27
Project development	Primary researchers	X	X	X																												
Ethical Committee approval	Primary researchers			X																												
Protocol preparation	Primary researchers	X	X	X																												
Usability study (US)		X	X	X	X	X	X	X	X	X	X	X	X	X	X	X	X	X														
Informed consent for US	Researchers responsible for US			X																												
Participants selection for US	Researchers responsible for US			X																												
*Focus group*	Researchers responsible for US			X																												
Users’ testing	Researchers responsible for US			X																												
Intermediate evaluation	Researchers responsible for US				X	X	X	X	X	X	X	X	X	X	X	X	X															
Incidence registration	Researchers responsible for US																	X														
Randomised controlled trial		X	X	X	X	X	X	X	X	X	X	X	X	X	X	X	X	X	X	X	X	X	X	X	X	X	X	X	X			
Interviewers’ training	Responsible for interviewers		X	X																												
Participants selection	Responsible for interviewers			X																												
Baseline evaluation	USAL			X																												
Treatment revision	Interviewers			X					X																							
Participants evaluation	Interviewers								X								X				X								X			
Randomisation	USAL/Sacyl/INTRAS			X																												
Intervention implementation	Primary researchers				X	X	X	X	X	X	X	X	X	X	X	X	X	X	X	X	X	X	X	X	X	X	X	X	X			
Data management and results		X	X	X	X	X	X	X	X	X	X	X	X	X	X	X	X	X	X	X	X	X	X	X	X	X	X	X	X	X	X	X
Database development	IBIP + USAL			X	X																											
Data collection	Interviewers				X				X								X				X								X			
Data analysis	Primary researchers + USAL														X	X	X	X	X	X	X	X	X	X	X	X	X	X	X	X	X	X
Data coding and upload	Primary researchers + USAL			X	X				X								X				X								X			
Hypotheses testing	Primary researchers + USAL								X	X	X	X	X	X	X	X	X	X	X	X	X	X	X	X	X	X	X	X	X	X	X	
Results and conclusions	Primary researchers				X	X	X	X	X	X	X	X	X	X	X	X	X	X	X	X	X	X	X	X	X	X	X	X	X	X	X	X
General project evaluation	Primary researchers	X	X	X	X	X	X	X	X	X	X	X	X	X	X	X	X	X	X	X	X	X	X	X	X	X	X	X	X	X	X	X
Dissemination		X	X	X	X	X	X	X	X	X	X	X	X	X	X	X	X	X	X	X	X	X	X	X	X	X	X	X	X	X	X	X
Dissemination of results (publications, conference presentations)	Primary researchers								X	X							X	X	X	X	X	X	X	X	X	X	X	X	X	X	X	X

*IBIP* Iberian Institute of Research in Psychosciences*, Sacyl* Health system in Castile and Leon, *USAL* University of Salamanca

### Analysis

Statistical analysis will be conducted using programme IBM SPSS v.23. A descriptive analysis of demographic and personal data will be conducted and results will be compared between all groups. Firstly, an analysis of normality and homogeneity of the data will be conducted. For normally distributed data, the repeated measures analysis of variance (ANOVA) will be used to examine the hypotheses in inter-subject and intra-subject analysis as well as in their interaction. This will be applied to all outcome measures in a comparative analysis of the data collected at from baseline, after 4 months and 12 months of the treatment. In case of non-homogeneous data, non-parametric methods will be used for analysis (Mann–Whitney *U* test, Kruskal-Wallis analysis of variance, Wilcoxon signed-rank test, calculation of Spearman’s correlation coefficient, etc.). Bonferroni/Dunnet corrections will be applied during post-hoc analysis if needed. The possible effects of randomisation bias will be analysed using linear-mixed models including within-group and between-group factors. The significance level will be set at *α* = .05.

Effect sizes and their respective standard errors (SE) and confidence intervals will be estimated as Standardised Mean Change Index (dMR) for within-group comparisons, and as Standardised Mean Index difference (dC) for between-group comparisons, and corrected for small sample sizes [54]:

### Exit strategy

A careful exit strategy is required due to the vulnerability of the participants, and may be required in the following situations:

1. The situation in which a participant decides to exit the study prematurely. Reasons for this could vary from sickness of the participant to lack of time to participate or even lack of interest in the project, etc.
  In this case, a questionnaire to explore the reasons for exit will be carried out. During the informed consent process, it will be clearly stated that a participant can leave at any time with no obligation to communicate the reasons for leaving. Therefore, the participant will be invited to voluntarily answer the questions of the exit questionnaire
  It will also be made clear to participants that leaving the project does not have any implications for the care that they receive and in a (voluntary) exit interview staff involved in the project will make sure that the project has come to a satisfactory closure and the participant feels comfortable with the situation
2. The situation in which the study period has come to an end.
  From the beginning of the pilot, it will be made clear to the participant that the study period is a maximum of 12 months of treatment and an additional 12 months of follow-up
  From the beginning of the study, it will also be clearly explained how the study will come to a close, ensuring participants are aware of the ending strategy and to prepare them for transition and closure
  If possible, options to continue using the GRADIOR and/or ehcoBUTLER platform under supervision of a mentor can be discussed (budget and time permitting)
  After closure of the study, the participant is expected to be able to use the ehcoBUTLER platform on their own. An information letter can be offered to answer the most common questions and to indicate a telephone number in case there are any questions after the study has ended

Neither study coordinators nor other members of staff can control or influence the interventions of participants in the TAU group. Therefore, participants with TAU can participate by their own initiative on other cognitive rehabilitation or stimulation programmes outside the competence of INTRAS Foundation.

## Discussion

We will conduct a RCT with a long-term cognitive rehabilitation intervention and follow-up. This innovative RCT aims to determine the influence of a long-term, computerised, cognitive rehabilitation programme on people with MCI and PwD in comparison to psychosocial stimulation and TAU. The preliminary usability study will focus on ensuring that this type of treatment is more convenient and beneficial for older people, as well as for healthcare professionals.

Limitations of the study are related to the long duration of the treatment along with the 1 year follow-up. Therefore, we expect a large number of participants to drop out due to loss of interest, medical reasons or worsening of a related health condition, changes in life situation, etc. This potential dropout rate has been considered in the sample size calculation to ensure the success of the trial. We also foresee the potential for interviewers and staff to drop out during the study. In this case, every new member of staff recruited will be informed about the study and every new interviewer will undergo the same training in evaluation tools as those recruited at the start of the study.

**Fig. 1:**
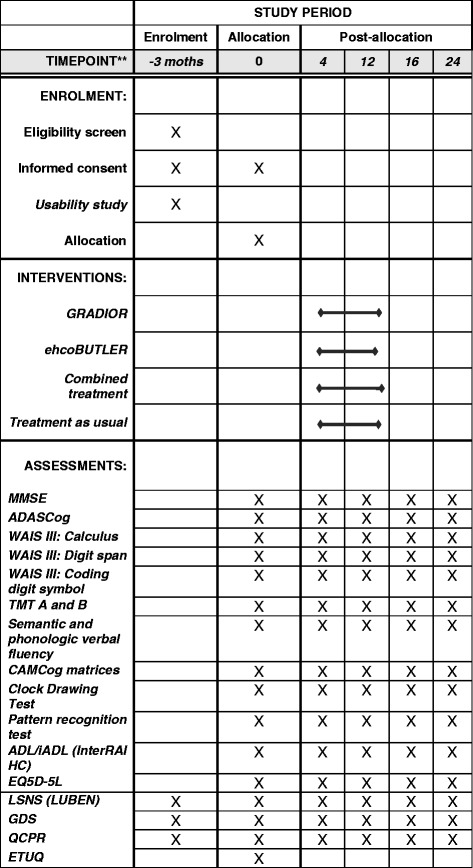
Standard Protocol Items: Recommendations for Interventional Trials (SPIRIT) Figure. Recommended items to address in a clinical trial protocol and related documents

For ethical and practical reasons, participants in this study will not be blinded to the intervention condition. Huntley et al. [55] recommend conducting single-blinded psychosocial intervention RCTs to minimise other factors, such as socialisation, when using the TAU control group (e.g. patient blinded and assessor blinded, but not therapist blinded). However, this is difficult to apply in practice due to ethical reasons. Therefore, we are conducting a single-blinded RCT with four parallel groups. In this study, all interviewers will be blinded to participants’ treatment allocation. Study coordinators who will manage and adjust the GRADIOR treatments of all participants will do so only based on data obtained from the programme and manage this under a participant’s code (Fig. 1 and Additional file 1).

## Trial status

The RCT trial on the effectiveness of GRADIOR started on 1 September 2016 and will be recruiting participants from 1 September 2017. The end of the trial is foreseen for 1 September 2019.

### Additional file

Additional file 1: SPIRIT Checklist. Recommended items to address in a clinical trial protocol and related documents. (DOC 120 kb)

## Abbreviations

ADASCog: Alzheimer’s Disease Assessment Scale – Cognitive Subscale
BDI-II: Beck Depression Inventory, 2nd edition
CAMCog: Cambridge Cognition Examination
CDR: Clinical Dementia Rating
COM: Combined treatment
dC: Standardised Mean Index difference
dMR: Standardised Mean Change Index
DSM-5: *Diagnostic and Statistical Manual of Mental Disorders*
EQ5D-5 L: EuroQoL 5 dimensions, 5 levels
ETUQ: Everyday Technology Use Questionnaire
GDS: Geriatric Depression Scale
GRA: GRADIOR
IBIP: Centro Ibérico de Investigación en Psicociencias (Iberian Research in Psychosciences Institute)
ICT: Information and communication technology
IESCYL: Instituto de Estudios de Ciencias de la Salud de Castilla y León (Study Institute of Health Sciences of Castile and Leon health system)
IWG: International Working Group
LSNS: Luben Social Network Scale
MCI: Mild cognitive impairment
MMSE: Mini Mental State Examination
PSS: Psychosocial stimulation
PwaMCI: People with amnestic MCI
PwD: People with dementia
QCPR: Quality of Patient-carer Relationship
QoL: Quality of life
RCT: Randomised controlled trial
Sacyl: Health system in Castile and Leon
SE: Standard error
STAI: State-Trait Anxiety Inventory
SUS: System Usability Scale
TAU: Treatment as usual
TMT: Trail-making test
USAL: University of Salamanca
WAIS-III: Wechsler Adult Intelligence Scale, 3rd edition
